# RNA sequencing as an alternative tool for detecting measurable residual disease in core-binding factor acute myeloid leukemia

**DOI:** 10.1038/s41598-020-76933-2

**Published:** 2020-11-18

**Authors:** TaeHyung Kim, Joon Ho Moon, Jae-Sook Ahn, Seo-Yeon Ahn, Sung-Hoon Jung, Deok-Hwan Yang, Je-Jung Lee, Myung-Geun Shin, Seung Hyun Choi, Ja-yeon Lee, Marc S. Tyndel, Hui Young Lee, Kyoung Ha Kim, Yu Cai, Yoo Jin Lee, Sang Kyun Sohn, Yoo Hong Min, June-Won Cheong, Hyeoung-Joon Kim, Zhaolei Zhang, Dennis Dong Hwan Kim

**Affiliations:** 1 Department of Computer Science, University of Toronto, Toronto, ON Canada; 2 The Donnelly Centre for Cellular and Biomolecular Research, University of Toronto, Toronto, ON Canada; 3 Department of Hematology-Oncology, Kyungpook National University Hospital, Daegu, Republic of Korea; 4 Department of Hematology-Oncology, Chonnam National University Hwasun Hospital, Hwasun, Jeollanam-do Republic of Korea; 5 Genome Research Center for Hematopoietic Disease, College of Medicine, Chonnam National University Hwasun Hospital, Hwasun-gun, Jeollanam-do Republic of Korea; 6 Department of Laboratory Medicine, Chonnam National University Hwasun Hospital, Hwasun, Jeollanam-do Republic of Korea; 7 The Edward S. Rogers Sr. Department of Electrical and Computer Engineering, University of Toronto, Toronto, ON Canada; 8 Department of Internal Medicine, Kangwon National University Hospital, Kangwon National University School of Medicine, Chuncheon, Gangwon-do Republic of Korea; 9 Department of Internal Medicine, Soonchunhyang University Hospital, Seoul, Republic of Korea; 10 Department of Hematology, Shanghai General Hospital, Shanghai Jiaotong University, Shanghai, People’s Republic of China; 11 Department of Internal Medicine, Yonsei University, Seoul, Republic of Korea; 12 Department of Molecular Genetics, University of Toronto, Toronto, ON Canada; 13 Department of Medical Oncology and Hematology, Princess Margaret Cancer Centre, Toronto, ON Canada

**Keywords:** Cancer genomics, Haematological cancer, Prognostic markers

## Abstract

DNA sequencing-based measurable residual disease (MRD) detection has shown to be clinically relevant in AML. However, the same methodology cannot be applied to fusion gene-driven subtypes of AML such as core-binding factor AML (CBF-AML). Here in this study, we evaluated the effectiveness of using DNA and RNA sequencing in MRD detection and in tracking clonal dynamics in CBF-AML. Using RNA-seq, we were able to quantify expression levels of *RUNX1*-*RUNX1T1* and *CBFB*-*MYH11* at diagnosis and their levels of reduction during remission (P < 6.3e−05 and P < 2.2e−13). The level of reduction of *RUNX1-RUNX1T1* as measured by RNA-seq and qPCR were highly correlated (R^2^ = 0.74, P < 5.4e−05). A decision tree analysis, based on 3-log reduction of *RUNX1*-*RUNX1T1* and c*KIT*-D816^mut^ at diagnosis, stratified *RUNX1-RUNX1T1* AML patients into three subgroups. These three subgroups had 2-year overall survival rates at 87%, 74%, and 33% (P < 0.08) and 2-year relapse incidence rates at 13%, 42%, and 67% (P < 0.05). On the other hand, although low residual allelic burden was common, it was not associated with long-term outcome, indicating that mutation clearance alone cannot be interpreted as MRD-negative. Overall, our study demonstrates that the clinical utility of RNA sequencing as a potential tool for MRD monitoring in fusion gene-driven AML such as *RUNX1-RUNX1T1* AML.

## Introduction

Leveraging on advances in next generation sequencing (NGS) technologies, a wide range of heterogenous genetic baseline characteristics has been discovered in acute myeloid leukemia (AML)^1,2^. Recent studies showed that targeted DNA sequencing-based measurable residual disease (MRD) detection is feasible in AML^3–7^. However, the weakness of targeted DNA sequencing based MRD detection is that it is not efficient to reliably track breakpoints of gene rearrangements using a targeted panel. The prevalence of gene rearrangement-driven AML is about 30%^8^. For instance, gene rearrangements affecting core binding factor (CBF) complex, *RUNX1*-*RUNX1T1*/t(8;21) or *CBFB*-*MYH11*/inv(16) are found in about 15% of AML patients (hereafter referred to as t(8;21) and inv(16) AML, respectively)^9^. Compared with other AML subtypes, CBF-AML is considered as a favourable risk subgroup due to favourable long-term outcomes following high-dose cytarabine-based chemotherapy^8,10^. However, up to 40% of CBF-AML patients still relapse^11–13^. As a post-remission monitoring of CBF-AML, a quantitative PCR (qPCR) assay targeting t(8;21) and inv(16) at remission can identify patients at high risk of relapse and has become a routine procedure^14–16^. Recently, a study by Dillon et al. demonstrated that MRD assessment using targeted RNA sequencing is feasible and correlates well with qPCR assays^17^.

While qPCR is highly sensitive and sequence-specific, it can only test a limited set of fusion events, making it neither scalable nor being used as a discovery tool. Thus, multiple tests have to be performed at diagnosis in order to characterize appropriate MRD targets to monitor post-treatment. In addition, standardization procedures make it challenging to compare qPCR results from different institutions and hospitals. On the other hand, RNA sequencing (RNA-seq) is scalable to quantify multiple targets in a single assay. In addition, it can also simultaneously quantify transcript expression, discover novel fusion transcripts, and even detect somatic mutations. Considering these advantages of RNA-seq and reductions in sequencing costs in recent years, we hypothesized that a single assay of RNA-seq can be utilized not only to detect mutations and fusion transcripts at diagnosis, but also quantify their reduction level at complete remission (CR).

Here we describe a study to evaluate DNA and RNA sequencing on longitudinal samples taken at diagnosis and at CR, as potential MRD detection tools for post-remission monitoring of CBF-AML. With this approach, we hoped to gain additional insights on how NGS can be applied to monitor MRD in CBF-AML, as well as reveal comprehensive dynamics of somatic mutations, transcripts, and gene rearrangements from diagnosis till CR. To address these questions, we conducted DNA sequencing (DNA-seq) on 223 DNA samples collected from 87 patients including 62 patients with t(8;21) and 25 patients with inv(16) taken at time points including diagnosis, CR, and relapse. For a subset of patients with available samples, we conducted RNA-seq on 90 samples consisted of 42 pairs of diagnosis-CR samples as well as 6 relapse samples.

The main goal of current study was to assess whether residual genetic alterations including primary (i.e. subtype-defining gene rearrangements) and secondary (i.e. single nucleotide variants and short indels) lesions at CR can be quantified using RNA and DNA-seq and are clinically relevant. Most notably, current study demonstrates how RNA-seq can be utilized to track gene rearrangements during remission and serves as a proof-of-concept study for RNA seq-based MRD detection for gene rearrangement-driven hematologic malignancies.

## Materials and methods

### Patient cohorts and acquisition of samples

Eighty-seven patients with newly diagnosed CBF-AML from January 2003 to September 2015 were included in this study. All patients had an Eastern Cooperative Oncology Group (ECOG) performance status ≤ 2 at the time of diagnosis. Patients treated with investigational drugs before induction therapy were excluded. Treatment and response assessments are summarized in Supplementary Information. Samples for NGS were obtained at the time of initial diagnosis (n = 87 and n = 42 for DNA and RNA-seq), CR (n = 53/87 and n = 42/42 for DNA and RNA-seq), and relapse (n = 15/87 and n = 6/42 for DNA and RNA-seq). CR samples were obtained either at day 28 (n = 45/53 and n = 34/42 for DNA and RNA-seq) or day 56 after re-induction (n = 8/53 and n = 8/42 for DNA and RNA-seq). Among 155 samples subjected for DNA sequencing, 151 samples were taken from bone-marrow (BM) and other 4 samples were taken from peripheral blood (PB). For patients with available serial samples, 90 samples (42 + 42 + 6) from 42 patients were also subjected to RNA-seq. All patient samples were collected after obtaining informed consent. Among 90 samples subjected for RNA sequencing, 86 samples were taken from BM and other 4 samples were taken from PB. All experiments and methods were carried out in accordance with relevant guidelines and regulations. This study was approved by the institutional ethics review boards at Chonnam National University Hwasun Hospital and Severance Hospital, Republic of Korea (CNUHH-2015-118).

### Sample preparation and next generation sequencing

BM/PB samples used in this study were prospectively procured and banked. After DNA isolation and library preparation, all samples were subjected to targeted sequencing. Targeted gene panel was constructed using a custom Agilent Sureselect design, covering exonic regions of 83 genes (Table S1 and Table S2). The gene list for targeted sequencing was compiled from our previous studies as well as large-scale mutation profiling studies on AML and MDS^1,2,18,19^. For RNA-seq, the total RNA was extracted from BM/PB mononuclear cells using the RNeasy mini kit (Qiagen, Germany). Total RNA concentration was calculated by Quant-IT RiboGreen (Invitrogen, Carlsbad, CA, USA). To assess the integrity of the total RNA, samples were run on the TapeStation RNA screentape (Agilent Technologies, Santa Clara, CA, USA). A total of 100 ng of total RNA was subjected to a sequencing library construction using a TruSight RNA Pan-Cancer Panel (1385 genes) (Illumina, Inc., San Diego, CA USA) according to the manufacturer's protocols. Both DNA and RNA samples were multiplexed and sequenced using an Illumina HiSeq 2500 (Illumina, San Diego, California, USA).

Sequencing metrics can be found in Table S3 (DNA-seq) and Table S4 (RNA-seq) and all sequencing data have been deposited at the European Nucleotide Archive (Accession: PRJEB25960 for DNA-seq and PRJEB27973 for RNA-seq). The mean on-target coverage for 223 DNA samples was 1,645.1X. RNA sequencing of 90 samples yielded an average of 3.6 million reads (range 2.8–4.5 M) with an average 86.9% mapping rate (range 83.4–90.5%). Read processing and variant calling procedures for DNA-seq data were performed as in our previous study with minor modifications^19^. Detailed methods on variant calling, RNA-seq read processing, transcript quantification, differentially expressed gene analyses and, fusion transcript discovery in diagnostic samples and its tracking in CR samples are provided in the Supplementary Information.

### Statistical analysis

Patient characteristics were summarized using descriptive statistics. Categorical variables were analyzed using a Chi-square test and comparison of continuous variables was performed using the Student’s t-test. The overall survival (OS) was defined as the time of diagnosis until death from any cause, which was analyzed using the Kaplan–Meier method. The groups were then compared using the log-rank test. The cumulative incidence of relapse was calculated using the Gray method, considering death without relapse as a competing risk. Non-relapse mortality was defined as any death while in remission with relapse as a competing risk and was assessed with the Gray’s test. The prognostic impact of risk factors on OS was determined using the Cox proportional hazard model. Fine-Gray proportional hazard regression with competing events were used to identify risk factors for relapse. P-values of less than 0.05 were considered significant. R statistical software 3.1.3 (the R foundation for Statistical Computing, Vienna, Austria; available at https://www.r-project.org) and EZR (version 1.33) were used to perform statistical analyses and generate all figures^20^.

## Results

### Patient characteristics and treatment outcomes

Among the 87 patients with newly diagnosed CBF-AML, 62 patients were t(8;21) AML and 25 were inv(16) AML. The median age of the patients was 44 years (range 16–85 years) and 50 patients (57%) were male. Induction chemotherapy was administered for 76 out of 87 (87%) patients. CR was achieved in 73 out of 76 patients (96%). The CR rate was 96% in both t(8;21) AML (n = 49/51) and inv(16) AML (n = 24/25). Three patients who failed to achieve CR died of treatment-related mortality (n = 2) or disease progression (n = 1). Post-remission therapy with high dose cytarabine was administered at CR. The patient characteristics are summarized in Table 1.

**Table 1:** Baseline clinical and genetic characteristics of 87 core binding factor acute myeloid leukemia patients enrolled in this study.

No. (%)	CBF-AML	*RUNX1-RUNX1T1*	*CBFB-MYH11*	P
Number of patients	87	62	25	
Age, median years (range)	44 (16–85)	43 (16–85)	47 (20–68)	0.55
**Gender**				
Male	50 (57)	37 (60)	13 (52)	0.68
Female	37 (43)	25 (40)	12 (48)	
WBC, × 10^9^/L, median (range)	16.9 (1.0–201.8)	10.4 (1.0–201.8)	40.1 (2.7–163.3)	0.52
**Mutations at baseline**				
*KIT*	34 (39)	28 (45)	6 (24)	0.11
D816	19 (22)	16 (26)	3 (12)	0.26
N822	13 (15)	13 (21)	0	0.02
*RAS*	36 (41)	19 (31)	17 (68)	< 0.01
*NRAS*	29 (33)	16 (26)	13 (52)	0.04
*KRAS*	11 (13)	5 (8)	6 (24)	0.10
*ASXL2*	12 (14)	12 (19)	0	0.03
Remission induction therapy	76 (87)	51 (82)	25 (100)	0.17
Achievement of CR, n = 76	73/76 (96)	49/51 (96)	24/25 (96)	1.00
Relapse, n = 73	19/73 (26)	13/49 (27)	6/24 (25)	1.00
Death	28 (37)	25 (40)	10 (40)	1.00

*CBF-AML* core binding factor acute myeloid leukemia, *WBC* white blood cell, *CR* complete remission.

### Targeted DNA sequencing reveals mutation profiles at baseline and their clinical relevance in CBF-AML

**Figure 1:**
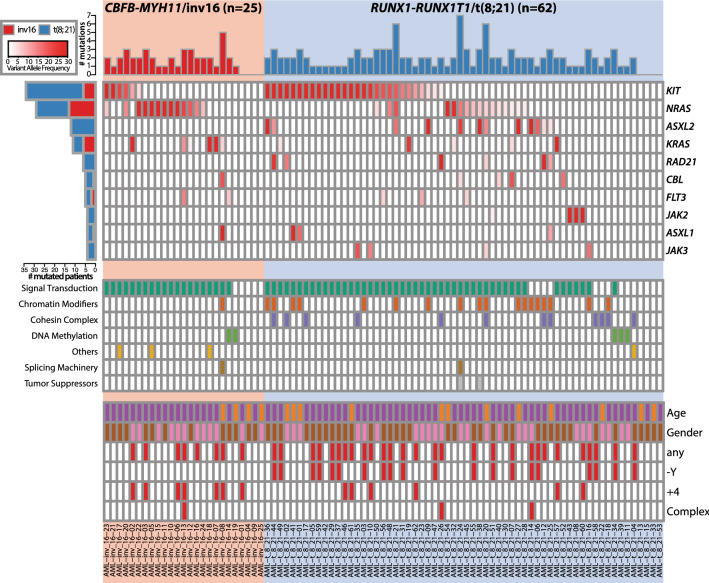
Spectrum of somatic mutation in 87 CBF-AML patients at initial diagnosis (n = 87). Targeted sequencing revealed 166 mutations at initial diagnosis of CBF-AML (62 t(8;21) AML and 25 inv(16) AML). The cohort is separated by their subtype of CBF-AML. inv(16) AML patients are shown on the left and t(8;21) AML patients are shown on the right. Each column indicates a single patient as well as each row represents a gene. Intensity of a cell in the heatmap on the top shows the relative level of VAF. In the heatmap on the top, only genes mutated more than 5% of the cohort are shown. The heatmap in the middle describes the presence of mutation in each of eight biological pathways (defined by TCGA). In the bottom, brief patient characteristics and notable cytogenetic information of each patient is shown.

At diagnosis, we detected 166 mutations in 79 patients (n = 79/87, 90.8%) (Fig. 1 and Table S5). The 166 mutations consisted of 120 nonsynonymous single nucleotide variants (SNVs), 21 frameshift insertions, 6 frameshift deletions, 5 non-frameshift insertions, 5 splicing events, 5 stop-gain SNVs, 2 non-frameshift deletions, and 2 synonymous SNVs. The median number of mutations per patient was 2 (range 1–7). Among the 83 targeted genes, 33 genes were mutated in at least one patient, and 18 genes were recurrently mutated. Notably, *KIT* (n = 34/87, 39%), *NRAS* (n = 29/87, 33%), *ASXL2* (n = 12/87, 14%), and *KRAS* (n = 11/87, 13%) were frequently mutated. Frequencies of mutation occurrence within targeted gene panel between two subtypes of CBF-AML, irrespective of genes, were comparable (93.5% in t(8;21) AML and 88% in inv(16) AML, p < 0.29). When grouped by associated biological pathways, genes associated with signaling pathways (n = 70/87, 80%), chromatin modifiers (n = 19/87, 22%) and, the cohesin complex (n = 13/87, 15%) were mutated in more than 10% of the cohort and frequencies of mutations in *RAS* (*KRAS* or *NRAS*), chromatin modifiers (including *ASXL2*) and, cohesin complex were significantly different between the two subtypes of CBF-AML (P < 0.002, P < 0.01, and P < 0.02, respectively, Figure S1). In particular, members of cohesin complex and chromatin modifiers were nearly exclusively mutated in t(8;21) AML.

We performed survival analyses to investigate association of somatic mutation with patient outcome (n = 76/87 after excluding the patients who did not receive chemotherapy) (Table S6). In univariate analysis, *KIT*-D816^mut^ was identified as an adverse prognostic factor for OS (Hazard ratio (HR) 2.31, 95% confidence interval (CI) 1.04–5.14, P = 0.035). Three-year OS rate was inferior in *KIT*-D816^mut^ group (47.1 ± 12.9%) to *KIT*-D816^wt^ group (68.6 ± 6.4%; P = 0.035). In terms of CIR (cumulative incidence of relapse), *KIT*-D816^mut^ was an adverse factor for CIR (HR 2.76 [1.06–7.15], P = 0.04), while mutations in *RAS* (*NRAS* or *KRAS*) was favorable for CIR (HR 0.13 [0.03–0.56], P = 0.01). Three-year CIR was higher in *KIT*-D816^mut^ group (43.8 ± 12.1%) than *KIT*-D816^wt^ group (22.0 ± 5.6%; P = 0.035). CIR was lower in *RAS*^mut^ subgroup than *RAS*^w*t*^ (6.4 ± 4.5% vs. 41.9 ± 7.8%; P = 0.001), while NRM (non-relapse mortality) was higher in *RAS*^mut^ subgroup (22.8 ± 7.5% vs. 7.3 ± 4.2%; p = 0.001). Thus the 3-year OS rate was not different between the groups of *RAS*^mut^ and *RAS*^w*t*^ (70.0 ± 8.3% vs. 59.0 ± 8.0%; P = 0.999). Overall, *KIT*-D816^mut^ was the only adverse prognostic mutation for OS.

### Longitudinal tracking of somatic mutations from diagnosis to remission and their clinical relevance

**Figure 2:**
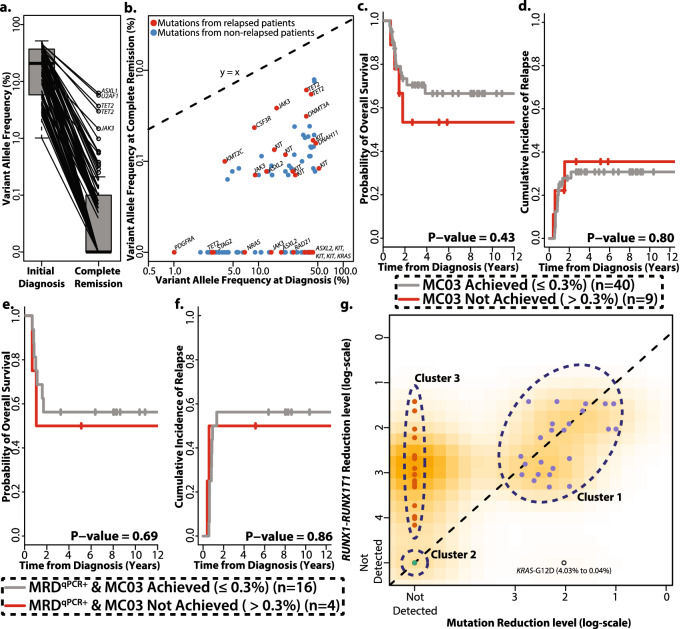
Dynamics and clinical relevance of somatic mutations during remission and its relationship with fusion transcript from serial sampling. (**a**) Reduction of allelic burden from diagnosis to CR. Ninety-nine mutations detected from 49 patients (4 patients without mutations at diagnosis) showed mean reduction rate of 99.1%. (**b**) Persistent allelic burden at CR and their association with relapse status. X-axis indicates VAF at initial diagnosis and Y-axis indicates VAF at CR. Red dots indicate mutations from relapsed patients and blue dots indicate mutations from non-relapsed patients. (**c**) Achievement of MC03 does not affect overall survival (HR 0.63, [0.20–1.97], P = 0.43) nor (**d**) relapse risk (HR 0.76, [0.23–3.04], P = 0.68). Among patients who were MRD-positive, achievement of MC03 does not affect (**e**) overall survival (HR 0.73, [0.15−3.52], P = 0.69) and (**f**) cumulative incidence of relapse. (HR 0.85, [0.14−5.14], P = 0.86). (**g**) Relationship between the reduction level of t(8;21) fusion transcript (measured by qPCR, y-axis) and VAFs (measured by NGS, x-axis). Each dot indicates a mutation, not a patient. Reduction level for both t(8;21) fusion transcript and VAFs were measured from the initial diagnosis to complete remission. Allelic burden was measured for each mutation and t(8;21) fusion transcript level assigned for each mutation is from the patient carrying the mutation. Color indicates their cluster assignment (cluster 1 as purple, cluster 2 as green, and cluster 3 as orange). Reduction level of allelic burden of cluster 1 and cluster 2 mutations is comparable to reduction level of mutation carriers’ t(8;21) fusion transcript level. Cluster 3 mutations show deeper reduction level compared to their carriers’ t(8;21) fusion transcript levels.

Taking advantage of samples taken at diagnosis and at CR, we assessed whether allelic burden at CR is clinically relevant. Among the 53 patients with available CR samples, 49 patients carried 99 mutations at diagnosis. Overall, 99 mutations showed mean reduction at CR with mean reduction rate of 99.1% (ranges from 86.2% to 100%, p < 0.0001). Out of 99 mutations, 53 mutations were cleared at CR (i.e. no reads supporting the variant allele) (Fig. 2a). The 46 detectable mutations at CR from 32 patients were commonly located in *KIT* (n = 13), *NRAS* (n = 8), and *KRAS* (n = 3). Mutations with higher VAFs at diagnosis are more likely to be detected at CR (mean VAF 27.4% for mutations detectable at CR vs. 17.8% for mutations cleared at CR, p = 0.001). To estimate mutation-like sequencing errors, we computed the frequency of mutation-like nucleotide changes in CR samples from patients who did not have the mutation in their diagnosis specimens. Overall, we observed that median mutation-like error rate is 0.008% in our data (interquartile range 0.004–0.013%). We then investigated whether the mutation clearance at CR is associated with subsequent occurrence of relapse (Fig. 2b). Among them, 15 mutations were from the 9 relapsed patients and 31 mutations were from the 23 non-relapsed patients. When comparing the frequency of mutation clearance, we did not find significant differences between the patients who relapsed and did not relapse (12/27 mutations in relapsed patients vs. 41/72 mutations in non-relapsed patients, p = 0.27). In addition, complete clearance rate in relapsed patients was comparable to that in non-relapsed patients (5/14 relapsed patients vs. 12/35 non-relapsed patients, P = 0.92). We then assessed clinical relevance of mutation clearance from various perspectives. Using 0.3% as a cut-off for allelic burden at remission, we did not find association of mutation clearance at 0.3% (MC03) with OS (HR 0.63, [0.20–1.97], p = 0.43) or with relapse risk (HR 0.84, [0.23–3.04], P = 0.80) (Fig. 2c,d). We have applied various other cut-offs but observed likewise patterns of no correlation with OS. For example, complete mutation clearance also did not any association with OS or relapse risk (HR 0.39, [0.11–1.36], p = 0.14 and HR 0.88, [0.32–2.47], p = 0.81). Among the 29 patients with *RUNX1*-*RUNX1T1* with available qPCR data, 20 patients were MRD-positive by qPCR at CR. Nine patients who achieved MRD-negative also achieved MC03 as well. When considering only MRD-positive patients, achievement of MC03 did not affect OS (HR 0.73, [0.15–3.52], P = 0.69) and relapse incidence (HR 0.85, [0.14–5.14], P = 0.86, Fig. 2e,f). Lastly, we assessed whether persistence of c*KIT*-D816^mut^ at CR is associated with higher risk of relapse, but complete clearance of *KIT*-D816^mut^ also did not affect OS and relapse incidence (HR 0.94, [0.16–5.18], P = 0.94 and HR 0.58, [0.16–2.09], P = 0.40, respectively, Fig. S2).

We next assessed the relationship between dynamics of the fusion transcript and mutation events. By comparing reduction level of the *RUNX1-RUNX1T1* fusion transcript measured by qPCR with reduction of allelic burden from 29 patients, we inferred the relationship between them (Fig. 2g). We defined three clusters showing distinct patterns. Cluster 1 consisted of mutations for which both somatic mutations and fusion transcript levels were detected (purple, 24 mutations from 17 patients). Cluster 2 was defined by both allelic burden and fusion transcripts not detected (green, 6 mutations from 3 patients). Cluster 3 was a group in which only somatic mutations were not detectable (orange, 28 mutations from 20 patients). Altogether, our results confirmed that the reduction level of fusion transcripts is more appropriate measure to assess MRD than single nucleotide variants and short indels in CBF-AML.

### Spectrum and dynamics of transcript expression in CBF AML from diagnosis to complete remission

**Figure 3:**
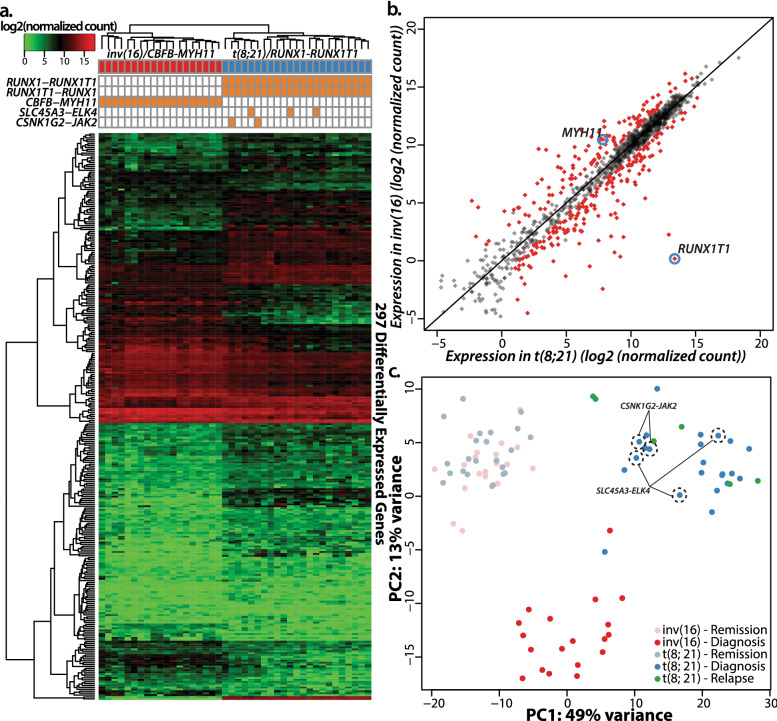
Spectrum and dynamics of targeted transcriptome in 42 CBF-AML patients. (**a**) Hierarchical clustering of 297 differentially expressed genes as well as summary of recurrent gene fusions. (**b**) Comparison of RNA expression between t(8;21) AML and inv(16) AML where *RUNX1T1* is the most differentially expressed genes between two related AML subtypes. (**c**) Principal component analyses reveal that two subtypes at diagnosis are distinctly clustered, whereas CR samples are clustered regardless of their subtype. In addition, patients with *SLC45A3*-*ELK4* (n = 3) and *CSNK1G2*-*JAK2* (n = 2) were unambiguously clustered with t(8;21) AML.

After reads processing and removing genes with very low expression in most samples, we quantified expressions of 1293 genes in 90 samples (Table S7)^21,22^. At diagnosis, we identified 297 differentially expressed genes (DEGs) between t(8;21) AML and inv(16) AML (Fig. 3a,b, Table S7, Fig. S3). Notably, *RUNX1T1* was the most differentially expressed gene between two subtypes, where t(8;21) AML expresses it nearly 4300 times higher than inv(16) AML (Fig. 3b). On the other hand, expression of *MYH11* was 6.3 fold higher in inv(16) AML (Fig. 3b).When paired diagnostic-CR samples for each subtype were compared, we found 402 and 286 DEGs in t(8;21) and inv(16) AML, where 200 genes were shared (Fig. S3, colored region). These 200 shared DEGs including *PD1*, *PD-L1*, *PD-L2*, and *CTLA4* were enriched in Kyoto Encyclopedia of Genes & Genomes (KEGG) terms related to immune response such as cytokine-cytokine receptor interaction (q < 2.2e−5), allograft rejection (q < 0.02) and, graft − versus − host disease (q < 0.02) (Figs. S3–S5, Table S8)^23^.

With respect to fusion transcripts, in addition to the subtype-defining fusion transcripts, we detected 26 additional fusion transcripts from 18 patients (9 inv(16) and 9 t(8;21) AML) including two recurrent fusion transcripts in t(8;21) AML *(SLC45A3*-*ELK4* and *CSNK1G2*-*JAK2*) (Fig. 3c and Table S9). Six samples taken at relapse (all t(8;21) AML) expressed *RUNX1-RUNX1T1* with breakpoints identified in their corresponding diagnostic samples and their transcriptome profiles were clustered with diagnostic t(8;21) AML samples (Fig. 3c). All CR samples were clustered together regardless of subtypes.

### Feasibility of RNA sequencing -based fusion transcript tracking as an alternative tool for MRD monitoring in CBF-AML

**Figure 4:**
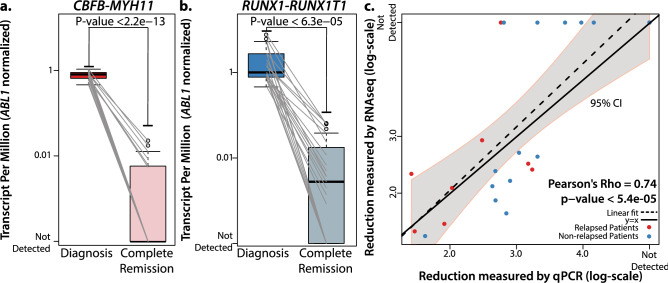
Tracking of *RUNX1-RUNX1T1* and *CBFB-MYH11*. Both fusion transcripts are detected reliably at diagnosis and show significant reduction at CR (P < 2.2e−13 and P < 6.3e−05, (**a**,**b**). (**c**) Comparison of *RUNX1-RUNX1T1* reduction levels measured by qPCR and RNA-seq. Results from qPCR and RNA-seq show positive correlation (Pearson’s Rho = 0.74, P < 5.4e−05).

Subtype-defining fusion transcripts were detected in all 42 diagnostic samples. In every CR sample, we tracked the fusion transcript identified in their corresponding diagnostic samples. As expected, both *RUNX1*-*RUNX1T1* and *CBFB*-*MYH11* showed significant reductions in all CR samples compared to their corresponding diagnostic samples (P < 6.3e−05 and P < 2.2e−13, paired t-test, Fig. 4a,b). Despite low expression, *CBFB*-*MYH11* was detectable in 6/19 (32%) and *RUNX1*-*RUNX1T1* was detectable in 15/23 (65%) CR samples. On average, 1618 reads were mapped to *RUNX1*-*RUNX1T1*(IQR 1284–1764 reads) and 239 reads were mapped to *CBFB*-*MYH11* (IQR 201–416 reads) in diagnostic samples. As the number of aligned reads were comparable between diagnostic and remission samples (3.01 M vs. 2.92 M reads, p = 0.19), limits of detection of *RUNX1-RUNX1T1* and *CBFB-MYH11* fusion transcripts using RNA-seq in CR samples would be approximately 3-log (1 in 1000) and 2-log (1 in 100) reduction. Reduction level of *RUNX1*-*RUNX1T1* measured by RNA-seq showed positive correlation with the reduction level measured by qPCR (Pearson’s Rho = 0.74, P < 5.4e−05, Fig. 4c and Table S10). Three-log or deeper reduction of *RUNX1-RUNX1T1* was able to predict relapse incidence (HR 0.22 [0.03–1.69], p = 0.14). When compared with qPCR-based MRD, RNA-seq-based MRD showed comparable performance in terms of sensitivity, specificity, positive/negative predictive value using 3-log reduction as a cut-off in *RUNX1-RUNX1T1* AML (Table S11). qPCR data were not available for CBFB-MYH11 AML. Survival analyses using mutation profile identified c*KIT*-D816^mut^ at diagnosis as an adverse prognostic factor for long-term outcome. Just as c*KIT*-D816^mut^ was all detected with high allelic burden using DNA-seq, it was reliably detected using RNA-seq as well (allelic burden ranges 20.8–46.4% in DNA-seq and 32.7–73.4% in RNA-seq) (Table S12).

### Prognostic modeling utilizing RNA-sequencing data in CBF-AML

**Figure 5:**
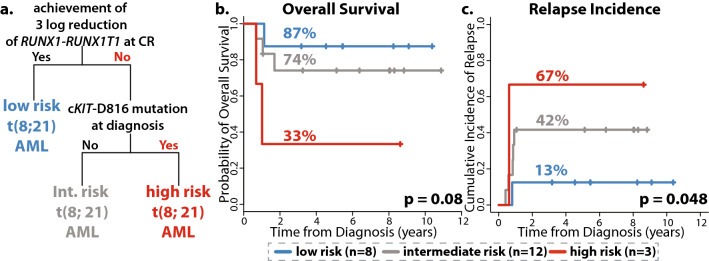
A prognostic model based on genetic features measured by RNA-sequencing. (**a**) A prognostic model using clinical and genetic information obtained from RNA-seq. Two variables were selected; 1. 3-log or deeper reduction of *RUNX1-RUNX1T1* transcript level and 2. presence of c*KIT*-D816 mutation at diagnosis. Based these two factors, the algorithm identified three subgroups. (**b**) Overall survival and (**c**) cumulative incidence of relapse based on the defined risk groups.

As RNA-seq can reliably measure reduction levels of fusion transcripts and detect high-risk mutations (i.e. c*KIT*-D816^mut^), we attempted to build a prognostic model using decision tree analysis incorporating genetic features obtained from RNA-seq. Decision tree analysis identified three distinct subgroups of *RUNX1-RUNX1T1* AML on the basis of reduction in the level of *RUNX1*-*RUNX1T1* transcript and mutation profile (Fig. 5a). Consistent with previous studies, 3-log or deeper reduction of *RUNX1*-*RUNX1T1* transcript was the most significant prognostic factor (low risk group)^14,15^. The algorithm further divided the patients who failed to achieve 3-log reduction according to the presence of c*KIT*-D816 mutation at diagnosis (intermediate and high-risk groups). For three defined groups, 2-year OS rates were 87%, 74%, and 33% (p = 0.08, Fig. 5b) and 2-year relapse incidence rates were 13%, 42%, and 67% (p = 0.048, Fig. 5c).

## Discussion

Current study demonstrated that RNA-seq can measure reduction levels of *RUNX1*-*RUNX1T1* and *CBFB*-*MYH11* from diagnosis to CR. *RUNX1-RUNX1T1* reduction levels measured by qPCR and RNA-seq showed high correlation and concordance. The prognostic mutation (i.e. *KIT*-D816^mut^) was reliably detected in both DNA and RNA-seq data. Solely using information from RNA-sequencing, we built a prognostic model that can predict long-term outcome for t(8;21) AML patients. On the other hand, complete clearance of secondary genetic lesions (i.e. single nucleotide variants and short indels) including *KIT*-D816^mut^ alone was not associated with better long-term outcomes and mutation persistence at low level did not provide prognostic power in addition to reduction level of the fusion transcripts. Altogether, our data and analyses suggest the clinical utility of RNA-seq as a method to detect MRD in CBF-AML and potentially in other fusion-driven hematologic malignancies.

Leveraging on paired-diagnosis-CR samples, we assessed the clinical relevance of residual genetic lesions at remission in CBF-AML. Although several studies demonstrated clinical relevance of residual allelic burden in non-CBF-AML, none of the studies thus far focused on CBF-AML^3–7^. To the best of our knowledge, transcriptome along with mutation profile on paired diagnostic-CR samples have not been investigated in CBF-AML. By tracking junctions identified in corresponding diagnostic samples, we were able to quantify the level of *RUNX1-RUNX1T1* and *CBFB-MYH11* in CR samples. The level of *RUNX1-RUNX1T1* reductions measured by RNA-seq was highly correlated with qPCR result (Pearson’s Rho = 0.74, p < 5.4e−05). *RUNX1-RUNX1T1* was detectable in 15 samples by both techniques and in 6 samples only by qPCR. In 2 samples, *RUNX1-RUNX1T1* was undetectable by neither method. qPCR was more sensitive at detecting trace amount of *RUNX-RUNX1T1* compared to RNA-seq at the sequencing coverage in our study (avg. = 2.98 M mapped reads per sample). However, we believe that these obstacles can likely to be overcome by achieving deeper sequencing depth as well as more tailored sequencing strategies. *CBFB-MYH11* was only detectable in 6/19 CR samples at the sequencing depth used in current study and qPCR results were not available for comparison. Future studies on *CBFB-MYH11* cohort with deeper sequencing depth and comparative analyses with qPCR remain to be investigated.

With respect to single nucleotide variants and short indels, allelic burden of all mutations showed significant reduction from diagnosis to CR (ranges from 86.2 to 100%). At CR, only 5 mutations out of 99 mutations (2 in *TET2*, 1 in *JAK3*, *U2AF1*, and *ASXL1* each) were observed with a VAF above 1%. This mutation clearance level is higher than what was reported in non-CBF-AML^3,4^. This could be due to differences in mutation profile between CBF and non-CBF-AML. Klco et al. only included intermediate and poor risk AML patients and most frequently persistent mutations were in DNA methylation or clonal hematopoiesis-associated genes (*DNMT3A*, *TET2*, *IDH1/2*)^3^. In CBF-AML, those genes are much less commonly mutated. Incorporation of mutation dynamics with *RUNX1-RUNX1T1* fusion transcript reduction level showed that reduction of mutation burden from diagnosis to CR is deeper than or equal to the reduction of *RUNX1-RUNX1T1* fusion transcript. This re-confirms that complete clearance of allelic burden (i.e. secondary lesion) during remission does not guarantee eradication of leukemic cells, but deeper reduction of primary lesion (i.e. fusion transcript) is a more appropriate surrogate marker for predicting relapse in CBF-AML. In addition to the assessment of the entire cohort with available CR samples, we also showed that residual allelic burden did not further stratify high risk patients among MRD-positive patients. This result indicates that DNA-based MRD detection, only targeting secondary lesions does not provide additional prognostic power in addition to reduction level of the fusion transcripts in CBF-AML.

Consistent with previous studies, mutation profiles of two CBF-AML subtypes were different^24–26^. For example, members of the cohesin complex and chromatin modifiers including *ASXL2* were nearly exclusively mutated in *RUNX1-RUNX1T1* AML. Genes associated with activated signaling such as *KIT*, *N/KRAS*, *CBL*, and *FLT3* were frequently mutated in both subtypes. Survival analyses showed that *KIT*-D816^mut^ was an adverse prognostic factor. As shown in Lavallée et al.^27^, the pattern of transcript expressions at diagnosis further distinguished these two related-subtypes of AML. CR samples were clustered regardless of their subtypes. We noticed that there are nine genes that are differentially expressed in CR samples depending on their AML subtypes. However, all nine genes were differentially expressed in at least one of three-way comparisons and this is likely due to the residual leukemia cells in CR samples.

There are several limitations in our study. First, the cohort size was relatively small, and the prognostic model developed using the current cohort could not be validated using an independent cohort. Second, the experimental setting of RNA sequencing utilized in this study such as the gene panel, amount of total RNA used, and sequencing depth was not optimized for MRD purposes. For example, detection of *CBFB-MYH11* using RNA-seq in CR samples requires experimental optimizations to be compatible with qPCR. Lastly, as some of fusion calls were only supported by few reads, further validation using alternative techniques such as qPCR should be performed to validate them.

Altogether, the current study demonstrates that RNA-seq can be utilized to monitor *RUNX1*-*RUNX1T1* and *CBFB*-*MYH11* level during remission in CBF-AML. Combining all of these supporting evidences, our data and analyses confirmed that the reduction level of fusion transcripts and baseline characteristics at initial diagnosis are the most important genetic features in CBF-AML when predicting long-term outcome and they provide complementary prognostic information. On the other hand, residual allelic burden of secondary lesion at CR measured by DNA-seq alone does not provide clinically relevant information in addition to those two genetic features. We also showed that a single assay of RNA-seq can be used to stratify *RUNX1*-*RUNX1T1* AML patients into three distinct risk groups according to their long-term prognosis. Our findings warrant prospective studies with larger cohorts on gene rearrangement-driven hematologic malignancies utilizing more tailored sequencing strategies.

## Supplementary information

Supplementary Information 1.

Supplementary Information 2.

Supplementary Information 3.

Supplementary Information 4.

Supplementary Information 5.

Supplementary Information 6.

Supplementary Information 7.

Supplementary Information 8.

Supplementary Information 9.
