# Supplementary material for: RNA sequencing as an alternative tool for detecting measurable residual disease in core-binding factor acute myeloid leukemia
Source: Sci Rep. 2020 Nov 18;10:20119. doi: 10.1038/s41598-020-76933-2 (PMC7674449; doi:10.1038/s41598-020-76933-2)
Supplement: Supplementary file 1 — Supplementary Information 1. [file 41598_2020_76933_MOESM1_ESM.docx]

**Supplementary Information**

**Title:** RNA sequencing as an alternative tool for detecting measurable residual disease in core-binding factor acute myeloid leukemia

**Authors**: TaeHyung Kim^1,2^, Joon Ho Moon^3^, Jae-Sook Ahn^4,5^, Seo-Yeon Ahn^4,5^, Sung-Hoon Jung^4,5^, Deok-Hwan Yang^4,5^, Je-Jung Lee^4,5^, Myung-Geun Shin^6^, Seung Hyun Choi^5^, Ja-yeon Lee^5^, Marc S. Tyndel^2,7^, Hui Young Lee^8^, Kyoung Ha Kim^9^, Yu Cai^10^, Yoo Jin Lee^3^, Sang Kyun Sohn^3^, Yoo Hong Min^11^, June-Won Cheong^11^, Hyeoung-Joon Kim^4,5*^, Zhaolei Zhang^1,2,12*^, and Dennis Dong Hwan Kim^13*^

^1^Department of Computer Science, University of Toronto, Toronto, Ontario, Canada ^2^The Donnelly Centre for Cellular and Biomolecular Research, University of Toronto, Toronto, Ontario, Canada ^3^Department of Hematology-Oncology, Kyungpook National University Hospital, Daegu, Republic of Korea ^4^Department of Hematology-Oncology, Chonnam National University Hwasun Hospital, Hwasun, Jeollanam-do, Republic of Korea ^5^Genomic Research Center for Hematopoietic Diseases, Chonnam National University Hwasun Hospital, Hwasun, Jeollanam-do, Republic of Korea, ^6^Department of Laboratory Medicine, Chonnam National University Hwasun Hospital, Hwasun, Jeollanam-do, Republic of Korea ^7^The Edward S. Rogers Sr. Department of Electrical and Computer Engineering, University of Toronto, Toronto, Ontario, Canada ^8^Department of Internal Medicine, Kangwon National University Hospital, Kangwon National University School of Medicine, Chuncheon, Republic of Korea ^9^Department of Internal Medicine, Soonchunhyang University Hospital, Seoul, Republic of Korea ^10^Department of Hematology, Shanghai General Hospital, Shanghai Jiaotong University, Shanghai, People’s Republic of China ^11^Department of Internal Medicine, Yonsei University, Seoul, Republic of Korea ^12^Department of Molecular Genetics, University of Toronto, Toronto, Ontario, Canada ^13^Department of Medical Oncology and Hematology, Princess Margaret Cancer Centre, Toronto, Ontario, Canada

* Corresponding author

**Treatment and response assessments**

Patients less than 65 years of age received induction chemotherapy of idarubicine 12 mg/m^2^ intravenous infusion for 3 days and cytarabine 200 mg/m^2^ intravenous infusion for 7 days. For elderly patients (≥ 65 years), supportive care such as transfusion and/or cytoreductive therapy was performed. Those who attained CR after one or two courses of induction therapy were treated with high dose cytarabine as consolidation therapy. High-dose cytarabine was given at a dose of 3 g/m^2^ every 12 hours on days 1, 3, and 5. The response assessment was conducted using the International Working Group response criteria for AML^1^.

**Cell sorting**

Cryo-preserved cells were thawed via addition of DMEM + 10% Fetal Bovine Serum (FBS). After thawing, cells were spun at 1,000 RPM for 5 minutes at 4℃. Thawing solution was then removed, and cells were re-suspended in 300 ul of Phosphate-buffered saline (PBS) + 2% FBS for exposure to antibodies. CD3-phycoerythrin (BD bioscience, California, USA) was used for cell sorting. Cell sorting was performed on the FACSAria III (BD bioscience, California, USA).

**Variant calling**

Sequencing reads were processed and somatic variants were called using the methodology described in our previous case study and a list of significant variants was generated for each case using 0.01 as a minimum variant allele frequency (VAF) in either diagnostic or relapse samples^2^. During the variant calling procedure, T-cell samples or samples taken at CR were used as controls for disease samples when calling variants for cases with available T-cell or CR samples. For patients with both T-cell and CR samples available, T-cell samples were used as a germline control. Within each case, the positions for all significant variants were checked at all available samples from the patients, since a variant found to be significant at one sample (e.g. diagnosis samples) might not necessarily have been found to be significant in the other (e.g. relapse sample), but its VAF at all stages would clearly be of interest. We backtracked and searched for occurrences of these variants in other samples within the cohort; the rationale being that the significance of a variant in any sample taken from CBF-AML patients increases the probability of it being significant in other CBF-AML patients where it is present. In addition, we ran the DeepSNV (R package) for all available samples using its default parameters to detect mutations that are present in control samples (T-cell or CR samples)^3^. Finally, we compiled a list of variants found to be significant in original TCGA study^4^ and searched for occurrences of them in our cohort that had not already been identified as significant.

Next, we attempted to identify and filter out variants likely to be single nucleotide polymorphisms (SNPs), germline mutations, or sequencing errors. We first discarded all variants with population frequency over 0.1% in any of NHLBI GO Exome Sequencing Project (ESP), 1000 Genomes Project or, The Exome Aggregation Consortium ^5-7^. For each variant that occurred in multiple cases with control samples, we searched for variants in which the control sample’s VAF is greater than a third of the tumour sample VAF. We removed these unless it occurred in a minimum of 25 cases in the COSMIC database (haematopoietic and lymphoid tissues only)^8^. For each of these variants, we also counted the total number of cases that met this condition. If this count reached 10% of the number of cases with control samples, the variant was removed from all cases (including occurrences that did not meet this condition). For variants that did not occur in a minimum of 5 haematopoietic and lymphoid tissue cases in the COSMIC70 database^8^, if they occur in any case with a germline VAF greater than a third of the tumour VAF, those variants were removed from all cases, regardless of the number of times they occurred within the cohort. The entire list of mutations after annotation using Annovar can be found in **Table S5**^5^.

**Real time quantitative polymerase chain reaction for measuring t(8;21) fusion transcript level**

For total RNA isolation, the RNeasy Plus Mini kit (Qiagen, Germany) was used according to the manufacturer’s instructions. Total RNA (1 ug) was reversely transcribed using the SuperScript III First-strand Synthesis System for RT-PCR (Invitrogen, California, USA). Real-time quantitative PCR was performed using Real-Q *AML1-ETO* Quantification Kit (Biosewoom, Korea) per described supplier’s instruction. Reactions were conducted on a 7500 Real Time PCR System (Applied Biosystems, California, USA). Each *AML1-ETO* fusion transcript level was normalized against the expression of *ABL1*. The absolute copy numbers of *AML1-ETO* and *ABL1* transcripts in the samples were calculated using fluorescence curves obtained from serially diluted standard cDNA. The results were expressed as the *AML1-ETO/ABL* ratio presented as a log-scale.

**Targeted RNA sequencing**

The total RNA was extracted from bone marrow/peripheral blood mononuclear cells using the RNeasy mini kit (Qiagen, Germany). Total RNA concentration was calculated by Quant-IT RiboGreen (Invitrogen, Carlsbad, CA, USA). To assess the integrity of the total RNA, samples are run on the TapeStation RNA screentape (Agilent Technologies, Santa Clara, CA, USA). A total of 100 ng of total RNA was subjected to a sequencing library construction using a TruSight RNA Pan-Cancer Panel (Illumina, Inc., San Diego, CA USA) according to the manufacturer's protocols.

The total RNA was firstly fragmented into small pieces using divalent cations under elevated temperature. The cleaved RNA fragments are copied into first strand cDNA using SuperScript II reverse transcriptase (Invitrogen Technologies, Carlsbad, CA, USA, #18064014) and random primers. This is followed by second strand cDNA synthesis using DNA Polymerase I, RNase H and dUTP. These cDNA fragments then go through an end repair process, the addition of a single ‘A’ base, and then ligation of the adapters. The products are then purified and enriched with PCR to create the cDNA library.

All libraries were normalized, and six libraries were pooled into a single hybridization/capture reaction. Pooled libraries were incubated with a cocktail of biotinylated oligos corresponding to coding regions of the genome. Targeted library molecules were captured via hybridized biotinylated oligo probes using streptavidin-conjugated beads. After two rounds of hybridization/capture reactions, the enriched library molecules were subjected to a second round of PCR amplification. The Captured libraries were quantified using KAPA Library Quantificatoin kits for Illumina Sequencing platforms according to the qPCR Quantification Protocol Guide (Kapa Biosystems, Wilmington, Massachusetts, USA) and qualified using the TapeStation D1000 ScreenTape (Agilent Technologies, Santa Clara, CA, USA). Indexed libraries were then submitted to an Illumina Hiseq 2500 sequencer (Illumina, Inc., San Diego, CA, USA), using the paired-end mode (2×101 bp)

**Targeted RNA sequencing read processing, transcript quantification, and differential gene expression analyses**

After quality control measures were confirmed using FastQC, we used tophat2 to map raw reads to human genome (hg19) using default parameter^9^. Gene-transfer file was downloaded from GENCODE (version 19) and filtered out genes which are not present in TruSight RNA Pan-Cancer Panel (Illumina, Inc., San Diego, CA USA) ^10^. After read mapping, transcript expression was analyzed initially using ht-seq followed by DEseq2 for down-stream analyses^11,12^. For differential expression analysis, we used minimum 0.05 as an adjusted p-value and 2-fold difference in transcript expression (normalized count) as a cut-off between pairs of subgroups (t(8;21) at diagnosis vs CR, inv(16) at diagnosis vs CR, and t(8;21) and inv(16) at diagnosis). Gene set enrichment analysis was performed using ClusterProfiler^13,14^.

**Discovery of fusion gene and longitudinal tracking of subtype-defining gene fusions**

EricScript was used to discover fusion gene in all RNA sequencing dataset with default setting and only high confidence calls (remaining in the filtered file) were included in the final list^15^. To track *RUNX1-RUNX1T1* and *CBFB-MYH11* in CR samples, we first compiled a list of recalibrated fusion transcripts from their corresponding diagnostic samples. We then combined the original transcript sequences used in EricScript with the list of fusion transcripts for read mapping. We used Burrow-Wheeler Aligner (version 0.7.17) to map both paired diagnostic-CR samples to compiled transcript sequences with default setting^16^. After mapping and sorting, potential PCR duplicates were removed using samtool’s rmdup command followed by filtering out reads with mapping quality 20 or less. We then quantified expression of fusion gene transcript using a transcript per million (TPM) for *RUNX1-RUNX1T1* and *CBFB-MYH11* in both diagnostic and CR samples, followed by normalization by expression of *ABL1* gene. TPM was computed as following:

1. For each transcript included in the TruSight RNA Pan-Cancer Panel and fusion transcripts identified in the diagnostic sample, the raw number of reads mapped to each transcript is divided by the transcript length.
2. Summation of normalized transcript expressions (computed at step 1) for all transcripts is further divided by one million.
3. Each normalized transcript expression (computed at step 1) is divided by the scaling factor (computed at step 2).

**Supplementary Figure legend**

**Figure S1.** Distinct mutation profile between two subtypes of CBF-AML. Gene and pathway-wise comparisons show that mutational landscape of CBF-AML with t(8;21) and inv(16) are distinct. **a.** gene-wise and **b.** pathway-wise comparison of mutation frequency between *CBFB*-*MYH11*/inv(16) and *RUNX1*-*RUNX1T1*/t(8;21) cohorts. **a.** gene-wise comparisons show that mutation frequencies in *RAS* (*N*/*KRAS*) and *ASXL2* are significantly different between two subtypes of CBF-AML (p = 0.0018 and p = 0.16, respectively) **b.** When grouped by well-defined biological pathway, chromatin modifiers and cohesin complex mutations were nearly exclusive to t(8;21) AML (p = 0.0098 and p = 0.016, respectively)

**Figure S2.** Kaplan-Meier curves for overall survival and cumulative incidence of relapse depending on the complete clearance of *KIT*-D816^mut^. **a.** Patients with complete clearance of *KIT*-D816^mut^ shows comparable overall survival (HR 1.00 [0.32 - 3.13] p=0.99) nor **b.** relapse risk (HR 1.14 [0.38 - 3.40], p = 0.81) compared to patients who had persistent *KIT*-D816^mut^.

**Figure S3.** Summary of three-way comparisons of differentially expressed genes. Out of 1293 genes, 524 genes are differentially expressed in at least one comparison.

**Figure S4.** Differential expressions of **a.** *PD1*, **b.** *PD-L1*, **c.** *PD-L2*, and **d.** *CTLA4* between diagnostic samples and CR samples.

**Figure S5.** Enriched KEGG terms for 200 differentially expressed genes that are shared between two subtypes of CBF-AML.

**Supplementary Tables**

**Table S1.** Annotation of mutated genes according to commonly mutated biological pathways in myeloid neoplasms

**Table S2.** List of genes subject for targeted DNA sequencing. The entire exonic regions of 83 genes were targeted covering about 327 kb.

**Table S3.** Sequencing metrics of 223 DNA samples sequenced in this study

**Table S4**. Sequencing metrics of 90 RNA samples sequenced in this study

**Table S5.** List and detailed annotations of all the mutations detected by the next generation sequencing

**Table S6**. Prognostic factors among patients treated with intensive induction therapy in univariate analysis (n=76)

**Table S7**. Expression profile of 1293 genes as well as the summary of differentially expressed genes in each comparison

**Table S8**. Enriched KEGG terms for differentially expressed genes shared between subtypes when compared corresponding CR samples.

**Table S9**. List of detected gene fusions in 42 diagnostic CBF-AML samples

**Table S10**. Expressions of *RUNX1-RUNX1T1*/t(8;21) and *CBFB-MYH11*/inv(16) measured by RNA sequencing and qPCR for diagnostic and CR samples

**Table S11**. Comparison of sensitivity, specificity, positive predictive value, and negative predictive value between RNA sequencing and qPCR

**Table S12**. Allele frequencies of c*KIT*-D816 mutations in DNA and RNA samples measured by RNA and DNA sequencing.

**References**

1. Cheson, B. D. *et al.* Revised recommendations of the International Working Group for Diagnosis, Standardization of Response Criteria, Treatment Outcomes, and Reporting Standards for Therapeutic Trials in Acute Myeloid Leukemia. *Journal of clinical oncology : official journal of the American Society of Clinical Oncology* **21,** 4642–4649 (2003).

2. Kim, T. H. *et al.* Clonal dynamics in a single AML case tracked for 9 years reveals the complexity of leukemia progression. *Leukemia* (2015). doi:10.1038/leu.2015.264

3. Gerstung, M., Papaemmanuil, E. & Campbell, P. J. Subclonal variant calling with multiple samples and prior knowledge. *Bioinformatics* **30,** 1198–1204 (2014).

4. Cancer Genome Atlas Research Network. Genomic and epigenomic landscapes of adult de novo acute myeloid leukemia. *N. Engl. J. Med.* **368,** 2059–2074 (2013).

5. Wang, K., Li, M. & Hakonarson, H. ANNOVAR: functional annotation of genetic variants from high-throughput sequencing data. *Nucleic Acids Res.* **38,** e164–e164 (2010).

6. 1000 Genomes Project Consortium *et al.* A global reference for human genetic variation. *Nature* **526,** 68–74 (2015).

7. Lek, M. *et al.* Analysis of protein-coding genetic variation in 60,706 humans. *Nature* **536,** 285–291 (2016).

8. Forbes, S. A. *et al.* COSMIC: exploring the world's knowledge of somatic mutations in human cancer. *Nucleic Acids Res.* **43,** D805–11 (2015).

9. Kim, D. *et al.* TopHat2: accurate alignment of transcriptomes in the presence of insertions, deletions and gene fusions. *Genome Biol.* **14,** R36 (2013).

10. Harrow, J. *et al.* GENCODE: the reference human genome annotation for The ENCODE Project. *Genome Res.* **22,** 1760–1774 (2012).

11. Anders, S., Pyl, P. T. & Huber, W. HTSeq--a Python framework to work with high-throughput sequencing data. *Bioinformatics* **31,** 166–169 (2015).

12. Love, M. I., Huber, W. & Anders, S. Moderated estimation of fold change and dispersion for RNA-seq data with DESeq2. *Genome Biol.* **15,** 31 (2014).

13. Yu, G., Wang, L.-G., Han, Y. & He, Q.-Y. clusterProfiler: an R package for comparing biological themes among gene clusters. *OMICS* **16,** 284–287 (2012).

14. Kanehisa, M. & Goto, S. KEGG: kyoto encyclopedia of genes and genomes. *Nucleic Acids Res.* **28,** 27–30 (2000).

15. Benelli, M. *et al.* Discovering chimeric transcripts in paired-end RNA-seq data by using EricScript. *Bioinformatics* **28,** 3232–3239 (2012).

16. Li, H. & Durbin, R. Fast and accurate long-read alignment with Burrows-Wheeler transform. *Bioinformatics* **26,** 589–595 (2010).
