# Supplementary figures and images for: RNA sequencing as an alternative tool for detecting measurable residual disease in core-binding factor acute myeloid leukemia
Source: Sci Rep. 2020 Nov 18;10:20119. doi: 10.1038/s41598-020-76933-2 (PMC7674449; doi:10.1038/s41598-020-76933-2)

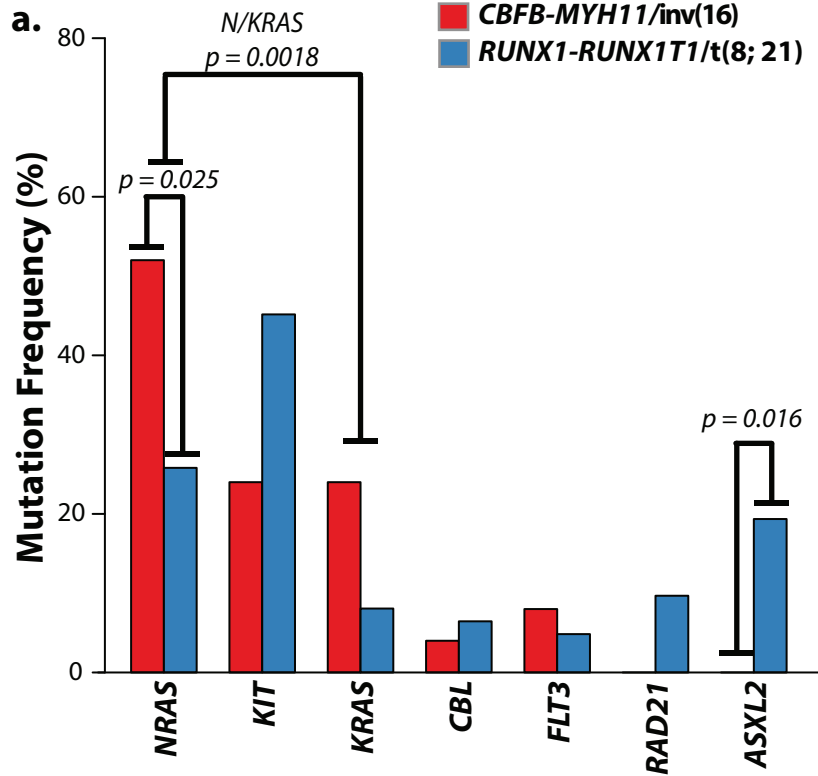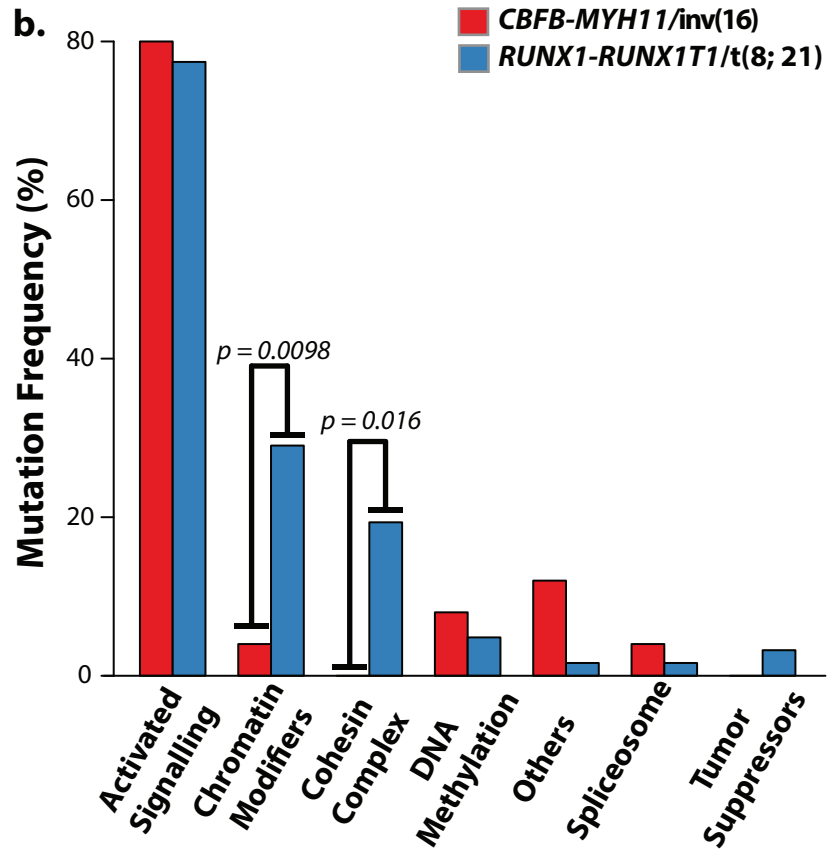

Supplement: Supplementary file 2 — Supplementary Information 2. [file 41598_2020_76933_MOESM2_ESM.pdf]

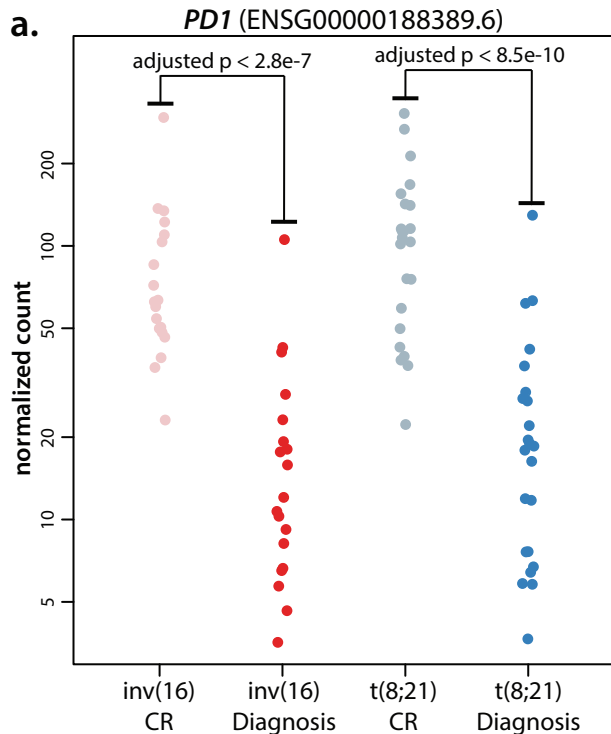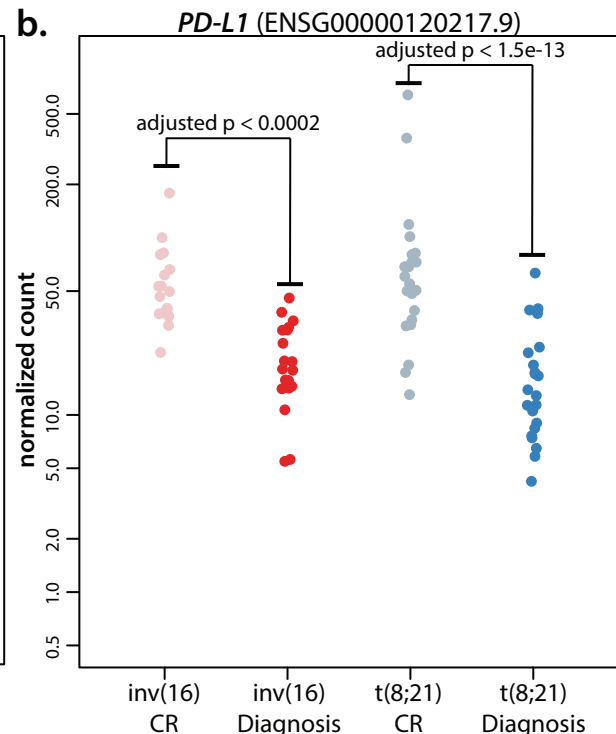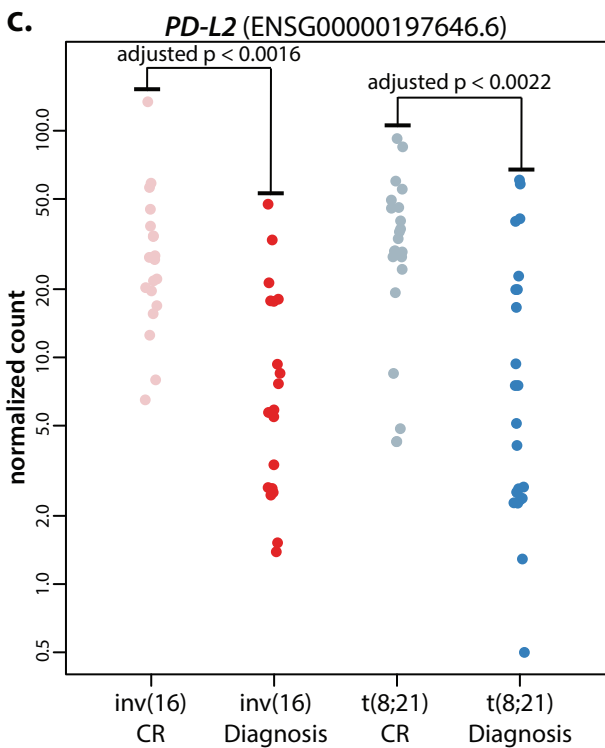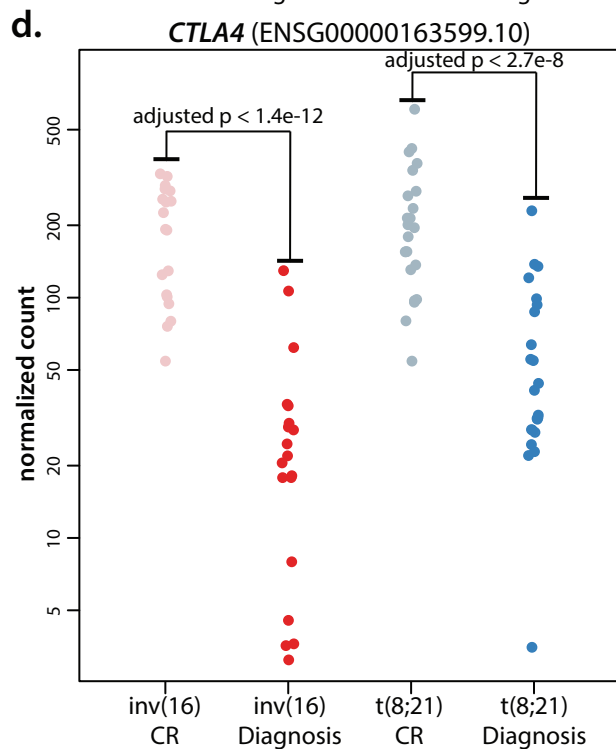

Supplement: Supplementary file 5 — Supplementary Information 5. [file 41598_2020_76933_MOESM5_ESM.pdf]
