# Supplementary material for: RNA sequencing as an alternative tool for detecting measurable residual disease in core-binding factor acute myeloid leukemia
Source: Sci Rep. 2020 Nov 18;10:20119. doi: 10.1038/s41598-020-76933-2 (PMC7674449; doi:10.1038/s41598-020-76933-2)
Supplement: Supplementary file 3 — Supplementary Information 3. [file 41598_2020_76933_MOESM3_ESM.pdf]

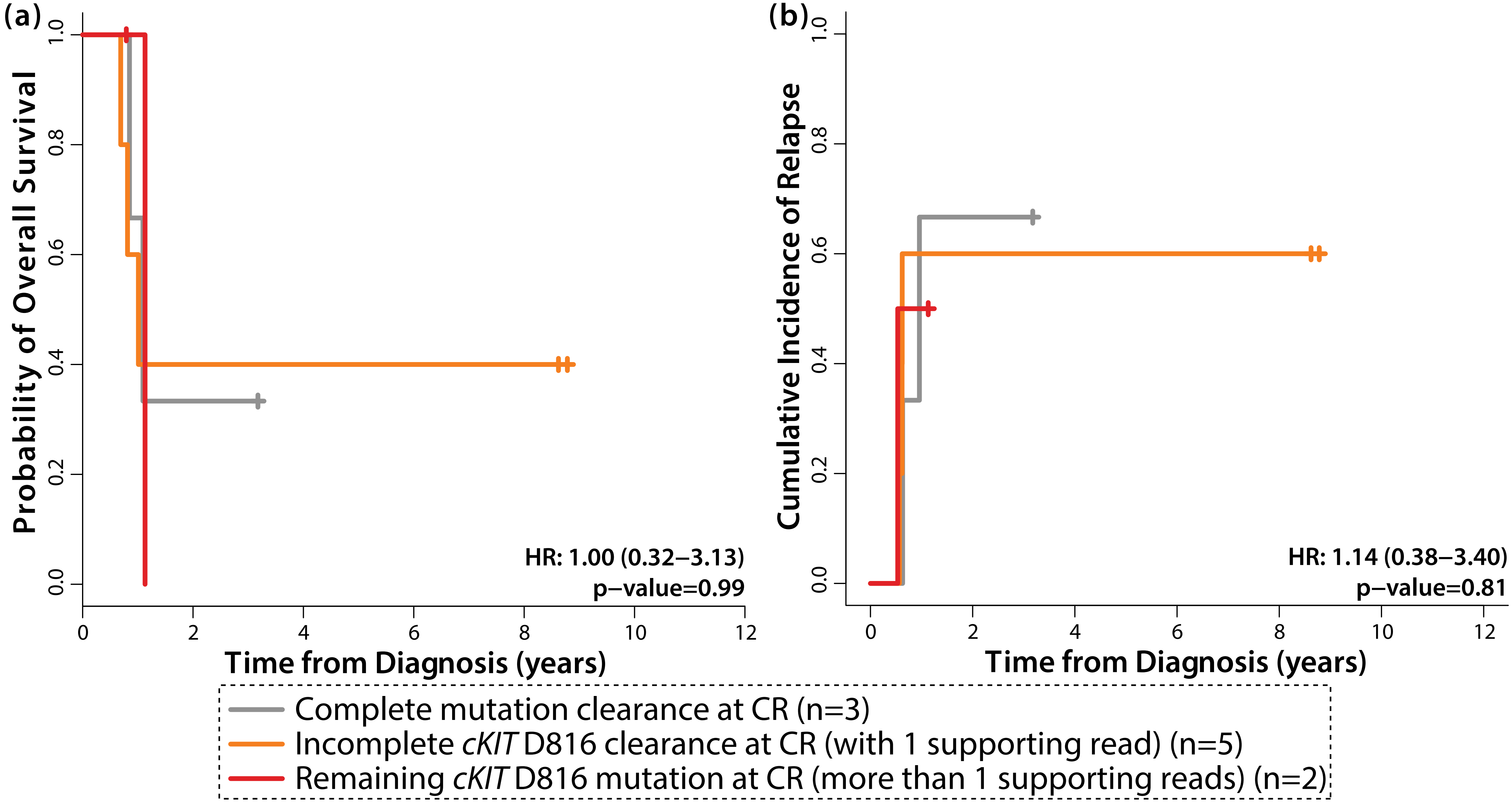

(a)
Probability of Overall Survival
1.0
0.8
0.6
0.4
0.2
0.0
HR: 1.00 (0.32–3.13)
p–value=0.99
0
2
4
6
8
10
12
Time from Diagnosis (years)
(b)
Cumulative Incidence of Relapse
1.0
0.8
0.6
0.4
0.2
0.0
HR: 1.14 (0.38–3.40)
p–value=0.81
0
2
4
6
8
10
12
Time from Diagnosis (years)
Complete mutation clearance at CR (n=3)
Incomplete *cKIT* D816 clearance at CR (with 1 supporting read) (n=5)
Remaining *cKIT* D816 mutation at CR (more than 1 supporting reads) (n=2)
