# Supplementary material for: RNA sequencing as an alternative tool for detecting measurable residual disease in core-binding factor acute myeloid leukemia
Source: Sci Rep. 2020 Nov 18;10:20119. doi: 10.1038/s41598-020-76933-2 (PMC7674449; doi:10.1038/s41598-020-76933-2)

Three-way comparisons of differentially expressed genes (1293 genes)

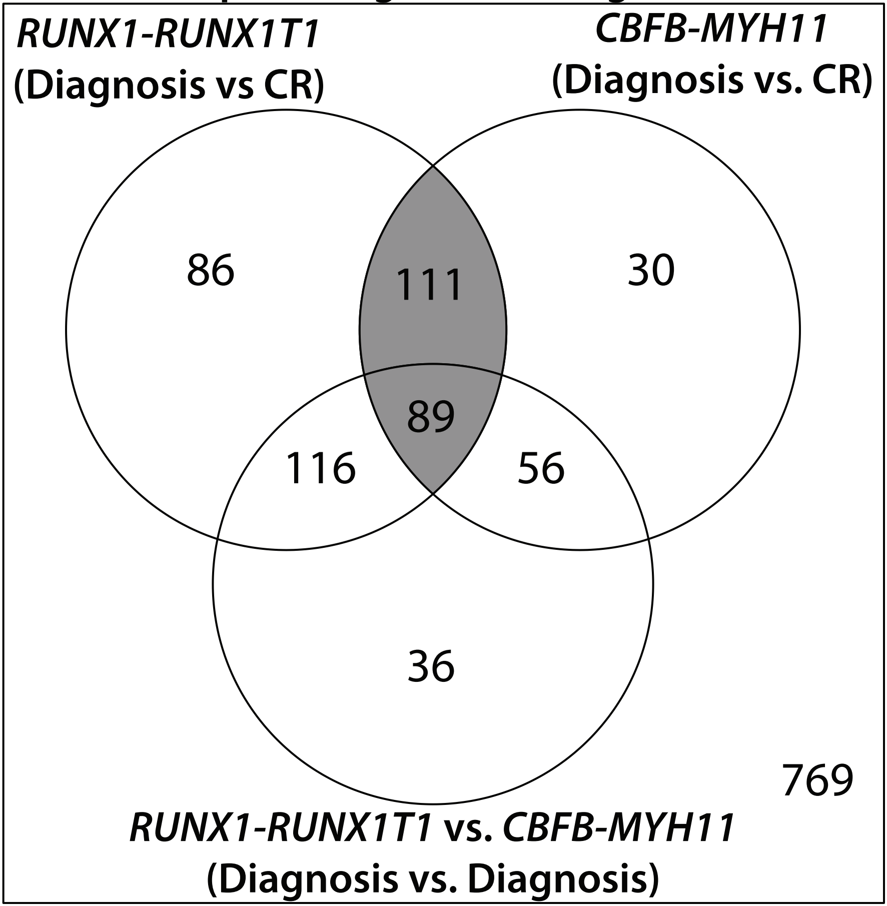

Supplement: Supplementary file 4 — Supplementary Information 4. [file 41598_2020_76933_MOESM4_ESM.pdf]
