# Supplementary material for: RNA sequencing as an alternative tool for detecting measurable residual disease in core-binding factor acute myeloid leukemia
Source: Sci Rep. 2020 Nov 18;10:20119. doi: 10.1038/s41598-020-76933-2 (PMC7674449; doi:10.1038/s41598-020-76933-2)
Supplement: Supplementary file 7 — Supplementary Information 7. [file 41598_2020_76933_MOESM7_ESM.docx]

| **Pathway** | **Genes** |
| --- | --- |
| Tumor suppressors | *PHF6, WT1* |
| DNA methylation | *DNMT3A, IDH2, TET2* |
| Signal Transduction | *CBL, CBLC, CDKN2A, CSF3R, FLT3, JAK1, JAK2, JAK3, KIT, KRAS, NOTCH1, NRAS, PDGFRA* |
| Splicing Machinery | *SF3B1, U2AF1* |
| Cohesin complex | *RAD21, SMC1A, SMC3, STAG2,* |
| Transcription factor | *FOXP1* |
| Chromatin modifier | *ASXL1, ASXL2, BCOR, CREBBP, EZH2, KDM6A, KMT2A, KMT2C* |
| Not used in pathway analyses | *DNAH11, DNAH5, MN1, SETBP1* |

**Table S1.** Annotation of mutated genes according to commonly mutated biological pathways in myeloid neoplasms
