# Supplementary material for: RNA sequencing as an alternative tool for detecting measurable residual disease in core-binding factor acute myeloid leukemia
Source: Sci Rep. 2020 Nov 18;10:20119. doi: 10.1038/s41598-020-76933-2 (PMC7674449; doi:10.1038/s41598-020-76933-2)
Supplement: Supplementary file 9 — Supplementary Information 9. [file 41598_2020_76933_MOESM9_ESM.docx]

**Table S6**. Prognostic factors among patients treated with intensive induction therapy in univariate analysis (n=76)

|  | Overall survival |  |  | Relapse |  |  | NRM |  |
| --- | --- | --- | --- | --- | --- | --- | --- | --- |
|  | HR (95% CI) | P |  | HR (95% CI) | P |  | HR (95% CI) | P |
| Age | 1.02 (0.99-1.04) | 0.30 |  | 1.03 (0.99-1.06) | 0.10 |  | 0.99 (0.95-1.03) | 0.74 |
| Gender, female vs male | 0.95 (0.45-2.04) | 0.90 |  | 0.67 (0.26-1.75) | 0.41 |  | 0.96 (0.32-2.88) | 0.94 |
| WBC ≥ 30.0 vs < 30.0* | 1.20 (0.56-2.56) | 0.64 |  | 0.98 (0.39-2.41) | 0.96 |  | 1.58 (0.54-4.60) | 0.40 |
| *CBFB-MYH11* vs  *RUNX1-RUNX1T1* | 1.27 (0.59-2.77) | 0.54 |  | 0.85 (0.34-2.18) | 0.74 |  | 2.04 (0.70-5.99) | 0.19 |
| Mutations |  |  |  |  |  |  |  |  |
| *KIT* D816^mut^ vs D816^wt^ | 2.31 (1.04-5.14) | 0.04 |  | 2.76 (1.06-7.15) | 0.04 |  | 1.09 (0.31-3.79) | 0.89 |
| *RAS*^mut^ vs *RAS*^wt^ | 0.99 (0.47-2.11) | 0.99 |  | 0.13 (0.03-0.56) | 0.01 |  | 5.10 (1.35-19.23) | 0.02 |
| *ASXL2*^mut^ vs *ASXL2*^wt^ | 0.47 (0.11-2.00) | 0.31 |  | 1.39 (0.41-4.75) | 0.60 |  | 0.48 (0.40-0.57) | 0.48 |

Abbreviations: HR, hazard ratio; CI, confidence interval; NRM, non-relapse mortality; WBC, white blood cell

*x10^9^/L
